# Effects of the subtypes of apolipoprotein E on immune inhibition and prognosis in patients with Hepatocellular Carcinoma

**DOI:** 10.1007/s00432-024-05856-6

**Published:** 2024-07-08

**Authors:** Bowen Gao, Peiyun Zhou, Li Wang, Zhutao Wang, Yong Yi, Xian Li, Jian Zhou, Jia Fan, Shuangjian Qiu, Yang Xu

**Affiliations:** 1 Department of Liver Surgery and Transplantation, Zhongshan Hospital, Key Laboratory of Carcinogenesis and Cancer Invasion of Ministry of Education, Liver Cancer Institute, Fudan University, Shanghai, 200032 China; 2 Shanghai Cancer Centre, Fudan University, Shanghai, 200032 China; 3 Institutes of Biomedical Science, Shanghai Key Laboratory of Medical Epigenetics, Shanghai Medical College, Fudan University, Shanghai, 200032 China

**Keywords:** Hepatocellular carcinoma, Apolipoprotein E, Myeloid-derived suppressor cells, Genetic factor, Prognosis

## Abstract

**Purpose:**

To investigate whether prognosis of patients with hepatocellular carcinoma (HCC) is affected by the abundance and subgroups of myeloid-derived suppressor cells (MDSCs) as well as subtypes and expression of apolipoprotein E (apoE).

**Methods:**

31 HCC patients were divided into three groups according to blood total apoE level for detecting the abundance of immunoregulatory cells by flow cytometry. Tumour tissue microarrays from 360 HCC patients were evaluated about the abundance and subgroups of MDSCs and the expression of apoE2, apoE3, apoE4 by immunofluorescence staining and immunohistochemistry staining. Survival analysis by means of univariate, multivariate COX regression and Kaplan-Meier methods of the 360 patients was performed based on clinical and pathological examinations along with 10 years’ follow-up data.

**Results:**

The lower apoE group presented higher abundance of MDSCs in the peripheral blood of HCC patients than higher apoE group. The abundance of monocyte-like MDSCs (M-MDSCs) was higher in the apoE low level group than high level group (*p* = 0.0399). Lower H-score of apoE2 (HR = 6.140, *p* = 0.00005) and higher H-score of apoE4 (HR = 7.001, *p* = 0.009) in tumour tissue were significantly associated with shorter overall survival (OS). The higher infiltration of polymorphonuclear granulocyte-like MDSCs (PMN-MDSCs, HR = 3.762, *p* = 0.000009) and smaller proportion of M-MDSCs of total cells (HR = 0.454, *p* = 0.006) in tumour tissue were independent risk factors for shorter recurrence-free survival (RFS).

**Conclusion:**

The abundance of MDSCs in HCC patients’ plasma negatively correlates with the level of apoE. The expression of apoE4 in HCC tissue indicated a poor prognosis while apoE2 might be a potential protective factor.

**Supplementary Information:**

The online version contains supplementary material available at 10.1007/s00432-024-05856-6.

## Introduction

Primary liver cancer (PLC) is a common cancer worldwide, with the sixth highest overall morbidity and the third highest mortality (Chidambaranathan-Reghupaty et al. 2021; Sung et al. 2021). HCC, which accounts for the largest proportion of PLC, presented with more than 900,000 new cases and 830,000 deaths in 2020 globally (Sung et al. 2021). The intrinsic biological characters of metabolic function, relatively tolerant immunological features and the prevalence of viral hepatitis have made the HCC an unique cancer for insight into the tumour biology (Hanahan 2022).

As a type of cancer occurs in liver, which is the metabolic centre of human body, the research of HCC could provide deep insight into the relationship between metabolism and cancer. Abnormalities in lipid metabolism have now been found not only in metabolic disorders but also chronic liver diseases such as hepatitis C virus (HCV) infection, alcoholic liver disease, etc. (Chidambaranathan-Reghupaty et al. 2021; Hu et al. 2020). The key molecule in the current investigation, apolipoprotein E (apoE), mainly synthesized by the liver, possesses a basic function as a composition of lipoproteins transporting lipid. Besides, the roles of apoE in immunoregulation (Tavazoie et al. 2018), lipid metabolic homeostasis and cell cycle(Du et al. 2020) and many other biological process have also been demonstrated. It participates in the biological processes of inflammatory pathology, oxidative stress (Li et al. 2022) and so on. Abnormal lipid metabolic pathways induce liver inflammation through processes such as lipid peroxidation, a molecular mechanism common to various chronic liver diseases contributing to similar cirrhotic outcome (Lawrence et al. 2019), which is a widely shared background for the oncogenesis of HCC, for the infection of HBV and HCV itself and the immune clearance could cause liver damage. Increasing frequency of genetic instability is shown in the process of proliferation and repairment of hepatocytes. In addition, immune cells including NK cells, Mucosal-associated-invariant-T lymphocytes (MAIT), NKT lymphocytes, cytotoxic T lymphocytes, T helper lymphocytes (Th), memory B lymphocytes and regulatory B lymphocytes (Bregs) in liver and relevant cytokines as well as chemokines contribute to the inflammation in various pathological conditions (Yuksel, Nazmi, et al., 2022). The persistence of chronic inflammations could cause microscopic histological abnormalities, along with the change of circulation state leading to severe destruction of liver parenchyma (Meng et al. 2021), and ultimately liver fibrosis(Goessling 2012; Yang et al. 2021).

Recently, although immunotherapies represented by anti-CTLA-4 antibodies and PD1/PD-L1 antibodies have made their debut in anti-cancer clinical practice(Xin Yu et al. 2020), the lack of universally accepted paradigm for the immune escape of cancer is one of the reasons limiting the effect of immunotherapies. To significantly improve the effect of immunotherapy for cancer, mechanism for immune escape of cancer is urgently needed. The immune tolerant nature of liver (Heymann and Tacke 2016) should be taken into account in the study of immune suppression and escape in HCC (Xin et al. 2022). The role of MDSCs in the current study, has received increasing attention in the immune escape mechanism of tumours in recent years(Zhao et al. 2023). MDSCs contain myeloid progenitors cells and immature myeloid cells released from the bone marrow, which then could be differentiated into immunosuppressive cell subpopulations in response to tumour-secreted chemokines and cytokines, and can be further classified into CD11b^+^CD14^−^CD15^+^CD66b^+^LOX1^+^ PMN-MDSCs and CD14^+^CD15^−^HLA-DR^−/low^ M-MDSCs (Gabrilovich and Nagaraj 2009). Existing studies have shown that MDSCs are highly involved in every stage of tumour and clinical outcomes (Cole et al. 2021).

A study showed that apoE could act on the LRP8 receptor on the surface of MDSCs, promoting their apoptosis and alleviating the suppressive effect of MDSCs on CD8^+^ T lymphocytes(Tavazoie et al. 2018). Another research in melanoma patients found that different apoE isoform allele carriers had significantly different tumour immunological biology and clinical outcomes, which was the first to give evidence that pre-existing genes in the genome can influence malignancy progression and patients’ prognosis (Ostendorf et al. 2020). However, melanoma happens in skin, which is an immunologically defensive and relatively aggressive organ, in contrary to the immune tolerant background of liver. Besides, lipid metabolism plays a much more important role in the development of HCC than in melanoma. Therefore, whether a similar phenomenon will be found in HCC remains unknown.

It is of interest how apoE, MDSCs and the relationship between them affect the progress and prognosis of HCC. In this study, we firstly performed flow cytometry on the peripheral blood samples from 31 patients treated with hepatectomy for HCC to seek the relationship between apoE and immune cells with suppressive phenotypes. We subsequently built a second cohort of 360 HCC patients to investigate how apoE subtypes, expression and infiltration of MDSCs in the HCC tissues affect patients’ survival, to examine its potential as a biomarker for HCC patients’ prognosis and as a therapeutic target. Other factors, especially lipid metabolism, were also analysed. We tried to construct a preliminary theoretical basis for the future application of apoE and MDSCs in clinic for diagnosis and treatment strategy selection, and in addition exploring the different biology of malignancies brought about by organ-specificity.

## Methods and materials

### Patients and samples

All patients were recruited from Zhongshan Hospital, Fudan University. The study was approved by the research ethics committee of Zhongshan Hospital (No. Y2020-374). Informed consent was obtained from all patients.

#### Cohort I

31 HCC patients were included, whose diagnosis was confirmed by the post-operation pathology. Their peripheral blood samples were collected.

#### Cohort II

360 cases were randomly selected from patients who underwent hepatectomy for liver neoplasm, whose HCC diagnosis was confirmed by post-operational pathology. The tumour tissues of these patients were collected. The clinicopathological information was extracted from hospital information system. Patients were followed up by healthcare professionals by telephone or using the outpatient medical system. The recurrence and/or metastasis of tumour were detected and determined by tumour biomarkers, abdominal ultrasound, x-ray computed tomography (CT) or magnetic resonance imaging (MRI) (once every 3–6 months, the frequency and methods of examination were determined according to the actual needs of the patient’s condition) to the end of the study or lost. OS was defined as the time from the date of surgery to the last follow-up visit or death, and RFS was defined as the time from the date of surgery to the date of tumour recurrence (intrahepatic recurrence or extrahepatic metastases). Patient records in this retrospective study cohort were established since 2012, with a follow-up end date of January 31, 2022.

### Flow cytometry

The patients in the first cohort were divided into high (> 53 mg/L)/normal (29-53 mg/L)/low (< 29 mg/L) groups based on the level of apoE in the peripheral blood according to the results of clinical laboratory examination. Flow cytometry was applied to evaluate the abundance of immune cells including MDSCs (anti-CD11b antibody: BioLegend, 101,228; anti-CD14 antibody: BioLegend, 325,622; anti-CD15 antibody: BioLegend, 125,604; anti-HLD-DR antibody: BioLegend: 307,642; anti-LOX antibody: BioLegend, 385,606; anti-CD33 antibody: BioLegend, 303,424), neutrophils (anti-CD15 antibody: BioLegend, 125,604), monocytes (anti-CD14 antibody: BioLegend, 325,622) and T lymphocytes (anti-CD3 antibody: BD, 564,001; anti-CD4 antibody: BioLegend, 344,616; anti-CD8 antibody: BioLegend: 301,041); typical immunosuppressive biomarkers, PD-1/PD-L1^+^(anti-PD-1 antibody: BioLegend, 329,920; anti-PD-L1 antibody: eBioscience, 12-5983-42) or CTLA-4^+^(anti-CTLA-4 antibody: eBioscience, 12-1529-42) were also evaluated. Two technical replicates were run for each blood sample. After being stained with surface antibodies and subsequently run on a FACS Aria III Flow Cytometer (BD Biosciences), data acquired were analysed by FlowJo software (Treestar) (Qiao and Luo 2019).

### Tissue microarrays construction

As was described in the previous reference, formalin-fixed paraffin-embedded (FFPE) samples were prepared, of which the cores of the tissue were extracted and arranged on blank receptors according to the tissue microarray design. Tissue cores were fused to the receptor paraffin blocks using a constant temperature oven (Roldan Urgoiti et al. 2014).

### Immunohistochemistry (IHC)

The paraffin sections were deparaffinised to water and heat-mediated antigen retrieval was carried out. After endogenous peroxidase blocked by 3% hydrogen peroxide solution and serum blocking using 3% BSA, primary monoclonal antibodies (apoE2: Absin, bs-4892r, 1:200; apoE3: Absin, bs-5039r, 1:200; apoE4: Absin, bs-5038r,1:200; ARG: Servicebio, GB11285, 1:200; iNOS: Servicebio, GB11119, 1:200) were added onto the slides. After being detected by HRP-labelled secondary antibody (Servicebio, GB23303, 1:200), DAB colour development solution (Servicebio, G1211) and hematoxylin (Servicebio, G1004) were used to stain the complex of antibodies and nuclei (Soiland et al. 2009). Hematoxylin-stained cell nuclei were blue and DAB revealed positive expression in brownish yellow. And then the slides were dehydrated and sealed for the following microscopic examination, image acquisition and high-throughput analysis by HALO (IndicaLabs), a digital pathology artificial intelligence analysis platform to derive an H-score for each sample(Isnaldi et al. 2020; Zwing et al. 2020).

### Immunohistofluorescence (IHF)

The paraffin sections were deparaffinised to water and heat-mediated antigen retrieval was carried out. After serum blocking, the slides were treated with primary monoclonal antibodies (CD11b: Servicebio GB11058, 1:200; LOX-1: Abcam: ab181470; HLA-DR: Abcam, ab92511) and fluorescent-labelled secondary antibody (CY3 conjugated Donkey Anti-Mouse IgG, Servicebio, GB21401/FITC conjugated Goat Anti-Rabbit IgG, Servicebio, GB22303). Nuclei was re-stained by DAPI (Servicebio, G1401). And then autofluorescence quencher (Servicebio, G1401) was added onto the slides for 5 min. Afterwards, the slides were sealed with anti-fluorescence quenching sealer. The slides were then observed under a fluorescent microscope (Nikon Eclipse C1) and images were taken (Nikon DS-U3, DAPI UV excitation wavelength 330–380 nm, emission wavelength 420 nm, blue light; FITC excitation wavelength 465–495 nm, emission wavelength 515–555 nm, green light; CY3 excitation wavelength 510–560 nm, emission wavelength 590 nm, red light) (Chu et al. 2019). The digital pathology artificial intelligence analysis platform HALO was applied to perform high-throughput analysis of the staining results of all the tissue microarrays, to derive the total number of cells in each sample, the count and percentage of target cells, and measuring the area of each sample.

### Statistics

Continuous variables were presented as “mean ± standard deviation” and categorical variables were presented as numbers or proportion, with p-values < 0.05 considered statistically significant by two-sided tests. Statistical analyses were performed using IBM SPSS Statistics 25 and R software (version 4.1.2). For variables which have been set up normal laboratory reference interval, the official ranges are used as cut-off in this research. For those without applied numbers, including IHC (apoE2, apoE3, apoE4, ARG and iNOS) and IHF (PMN-MDSCs and M-MDSCs) results, we applied x-tile software to screen the cut-off values of them, using OS or RFS respectively. After a univariate COX regression analysis of survival data with 0.1 as the test introduction level, a multivariate COX regression analysis was performed. Variables with p-values < 0.05 in the COX regression analysis and those that were closely related to this study or clinically significant were included in the multivariate model and expressed in terms of hazard ratio (HR) and 95% confidence interval (CI). ANOVA and T-tests were used to compare the difference among groups in flow cytometry, p-values < 0.05. Statistical plots were drawn using Graphpad Prism software (version 9.4.1).

## Results

### Relevance between the level of apoE and the abundance of immune suppressor cells in peripheral blood of HCC patients

#### The relevance between the levels of apoE in plasma and phenotypes/abundance of MDSCs in peripheral blood

**Fig. 1:**
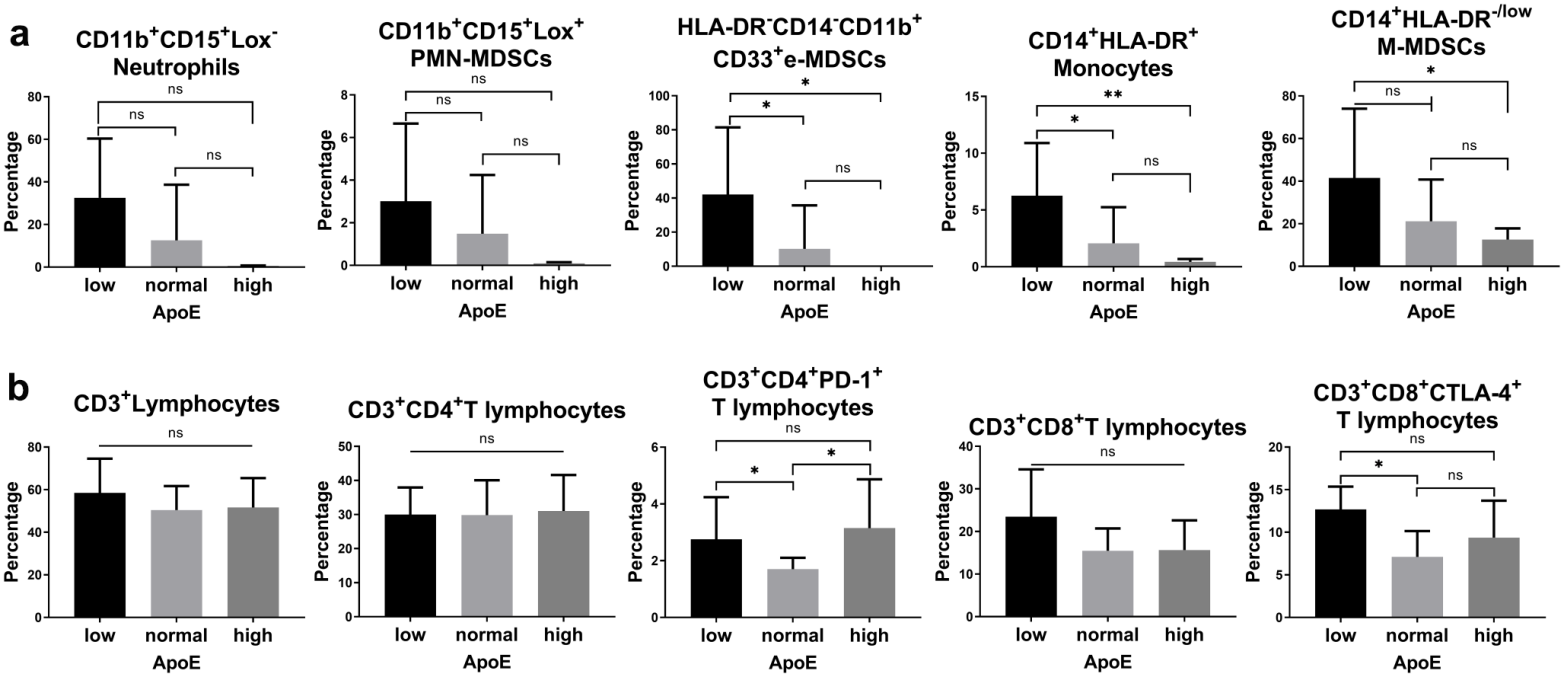
**a**. The abundance of MDSCs and associated immune cells and the subgroups with expressions of immune suppressor biomarkers in HCC patients with low, normal or high levels of apoE. **b**. The abundance of CD3^+^CD4^+^ T lymphocytes, CD3^+^CD8^+^ T lymphocytes and the subgroups with expressions of immune suppressor biomarkers in HCC patients with low, normal or high level of apoE

Two-by-two comparisons were made between three subgroups of low, normal and high level of plasma apoE. Firstly, analysis in HCC patients suggested that CD14^+^HLA-DR^-^ M-MDSCs (*p* = 0.0399) was significantly higher in the group with low levels of apoE than in the high level apoE group; the abundance of CD11b^+^CD33^+^CD14^-^HLA-DR^-^ e-MDSCs was significantly higher in the low level apoE group than in the normal level apoE group (*p* = 0.0327) and the high level apoE group (*p* = 0.0156). The abundance of CD14^+^HLA-DR^+^ monocytes was significantly higher in the low level apoE group than that in normal level group (*p* = 0.0223) and high level apoE group (*p* = 0.0065) (Fig. 1a and Supplementary Fig. 1).

#### The relevance between the levels of apoE in plasma and phenotypes/abundance of immunosuppressive T lymphocytes in peripheral blood of HCC patients

No statistically significant differences were observed in the abundance of CD3^+^ lymphocytes (T lymphocytes) in peripheral blood among the groups of HCC patients with different levels of apoE.

Further analysis revealed significantly higher abundance of CD3^+^CD4^+^PD-1^+^ T lymphocytes in the groups of patients with low level apoE (*p* = 0.0291) and high level apoE (*p* = 0.008) than in the group with normal level apoE, but no significant differences were observed between the of groups of high level apoE and low level apoE. No statistically significant differences were observed in the abundance of CD3^+^CD4^+^CTLA-4^+^ and CD3^+^CD4^+^PD-L1^+^ T lymphocytes among the groups. T lymphocyte abundance of CD3^+^CD8^+^CTLA-4^+^ was significantly higher in the low level apoE group than in the normal level apoE group (*p* = 0.0115), and no statistically significant differences were observed in the results of the two-way comparison between the CD3^+^CD8^+^PD-1^+^ and CD3^+^CD8^+^PD-L1^+^ T lymphocyte abundance groups (Fig. 1b and Supplementary Fig. 2).

**Fig. 2:**
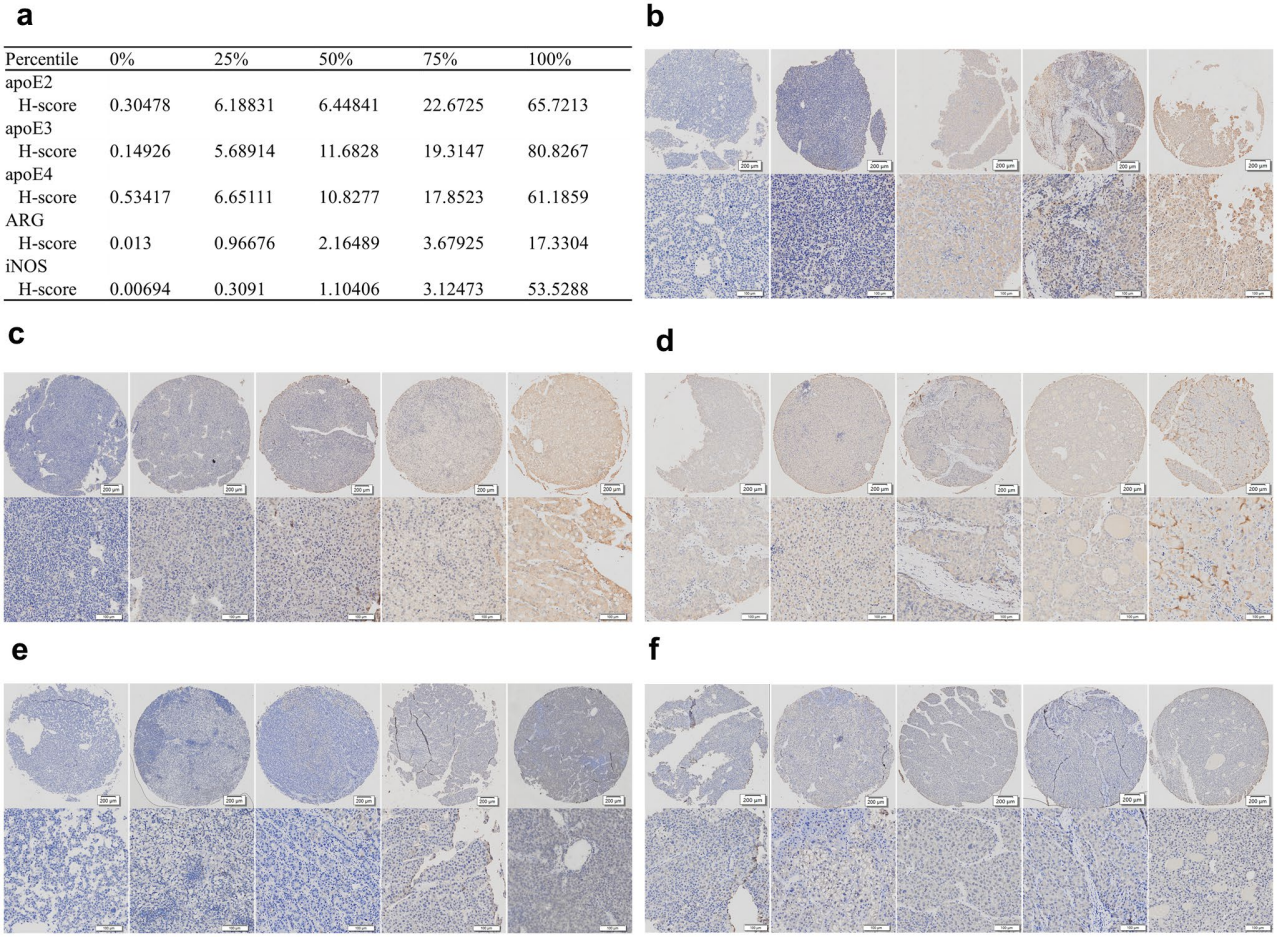
The representative views of IHC staining. (**A**) The ranking, percentile and H-score of the immunohistochemical staining results, correspond to B, C, D, E and F in Fig. 2. (**B**) IHC staining of apoE2: the area and depth of the brown staining indicated the expression of target protein. These are representative views of the IHC acquired by scanning and prepared for the following analysis by HALO software. The other IHC figures are being presented in the same way

### Evaluating the apoE expression in HCC tissues

To determine the subtypes and to evaluate expression of apoE in HCC tissues, IHC staining was performed (Fig. 2b and f). The ranking and percentile of the H-scores for each sample in this study cohort are shown in Fig. 2a.

### Detecting the infiltration of MDSCs in the HCC tissues

**Fig. 3:**
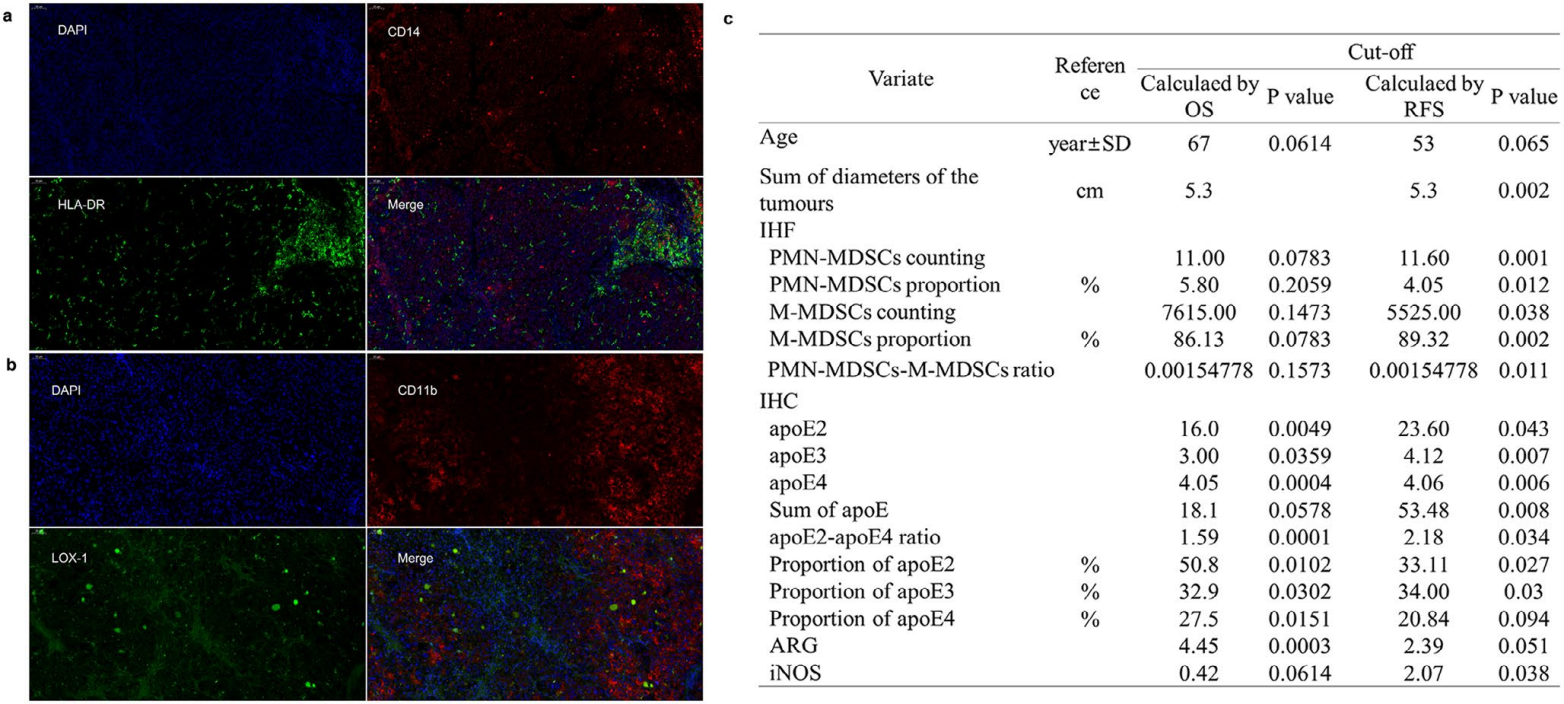
Expressions of CD14/HLA-DR and CD11b/LOX-1 in tissue microarrays by IHF analysis. **A** & **B**. DAPI staining in cell nucleus, blue, x100; CD14 and CD11b expression on the cell membrane, red, x100; HLA-DR and LOX-1 expression on the cell membrane, green, x100. Merged figures of expression of CD14 and HLA-DR, single positive for CD14 exhibiting PMN-MDSCs for further processing in HALO software. Merged figures of double-positive expression of CD11b and LOX-1 exhibiting M-MDSCs for further processing in HALO software. C. Cut-off values calculated by x-tile software

IHF staining was applied to detect the infiltration of MDSCs in HCC tissues (Fig. 3a and b). Cells with CD11b^+^LOX1^+^ (double positive) were PMN-MDSCs, and cells with CD14^+^HLA-DR^−/low^ (single positive) were M-MDSCs.

### The statistics of the clinical and experimental characteristics and survival analysis of cohort II

The clinical and experimental pathological characteristics of the 360 patients were collected and analysed, as presented in Table 1. The results for the screening of the cu-off values are shown in Fig. 3c.

**Table 1:** The statistical description of the clinical and experimental characteristics of cohort II

Variate	Reference	Mean±SD/count/proportion	Reference intervals for clinical laboratory tests at Zhongshan Hospital, Fudan University
Age	year	54.3 ± 10.98	
	≤ 67/>67	39/319	
Gender	female/male	50/310	
Liver disease backgrounds			
Virus hepatitis			
HBsAg	-/+	42/315	
HBVDNA	50IU/mL	65161.47 ± 210997.77	Real-time PCR with lower limit: 50IU/mL
	-/+	188/153	
Liver cirrhosis (pathology)	-/+	80/269	
G score	0/1, 2/3, 4	26/288/38	
S score	0/1, 2/3, 4	45/93/214	
Liver cirrhosis nodules	cm	0.35 ± 0.2	
Steatosis (pathology)	-/+	258/102	
Tumour pathology			
Tumour counting	1/>1	290/67	
Sum of diameters of the tumours	cm	5.07 ± 2.98	
	≤ 5.0cm/>5.0cm	217/129	
Grade of differentiation	High/Medium/Low	8/301/47	
Tumour capsule	Complete/None or uncomplete	232/121	
Gross tumour emboli	-/+	350/9	
Microscopic tumour emboli	-/+	223/133	
Laboratory examination			
Liver function test and metabolism			
Protein metabolism: Albumin	g/L	40.32 ± 3.06	35–55
	< 35/35–55/>55	0/344/15	
Bilirubin metabolism test			
TB	µmol/L	11.6 ± 5.06	3.4–20.4
	< 3.4/3.4–20.4/>20.4	0/334/25	
DBIL	µmol/L	4.66 ± 1.88	0.0-6.8
	≤ 6.8/>6.8	299/46	
Enzymology test			
ALT	U/L	36.37 ± 24.04	7–40
	< 7/7–40/>40	2/238/117	
AST	U/L	35.75 ± 17.61	13–35
	< 13/13–35/>35	1/206/138	
γ-GT	U/L	69.62 ± 62.75	7–45
Glycometabolism: blood glucose	mmol/L	5.46 ± 1.55	3.9–5.6
	< 3.9/3.9–5.6/>5.6	4/255/88	
Coagulation function			
PT	s	12.32 ± 0.8	10.0–13.0
	< 10.0/10.0–13.0/>13.0	0/290/69	
INR		1.06 ± 0.07	0.50–1.20
Lipid metabolism			
Triglyceride(TG)	mmol/L	1.17 ± 0.58	< 1.70
	< 1.70/≥1.70	288/46	
Cholesterol (CHO)	mmol/L	4.21 ± 0.92	< 5.20
	< 5.20/>5.20	295/39	
HDL	%	1.28 ± 0.38	> 1.04
	> 1.04/≤1.04	240/94	
N-HDL	mg/L	2.93 ± 0.88	< 4.10
	< 4.10/≥4.10	302/32	
LDL	mmol/L	2.4 ± 0.8	< 3.40
	< 3.4/≥3.4	305/29	
apoA I	g/L	1.18 ± 0.27	1.0-1.9
	< 1.0/1.0-1.9/>1.9	80/250/4	
apoB	g/L	0.75 ± 0.22	0.75–1.50
	< 0.75/0.75–1.50/>1.50	181/152/1	
apoE	mg/L	39.82 ± 12.6	29–53
	< 29/29–53/>53	65/225/44	
LPA	ng/mL	176.95 ± 226.89	0-300
	≤ 300/>300	279/55	
Liver fibrosis marker: type III procollagen	ng/mL	12.47 ± 9.76	< 15
	≤ 15/>15	257/79	
Tumour biomarkers			
AFP	ng/mL	2944.34 ± 9484.39	< 20.0
	< 20/≥20	168/191	
	< 400/≥400	265/94	
CA19-9	U/mL	23.07 ± 57.02	< 34
	< 34/≥34	283/43	
CEA	ng/mL	2.73 ± 1.78	< 5
	< 5/≥5	304/24	
CRP	/uL	4 ± 10.53	0.0–3.0
	≤ 3.0/>3.0	243/78	
IHF (byH-score)			
PMN-MDSCs counting		201.44 ± 587.19	
PMN-MDSCsproportion	%	11.01 ± 19.67	
M-MDSCs counting		7556.24 ± 5477.21	
M-MDSCs proportion	%	64.11 ± 37.74	
PMN-MDSCs-M-MDSCs ratio (PMN-MDSCs/M-MDSCs)		2.06 ± 29.98	
IHC (byH-score)			
apoE2		15.50 ± 12.74	
apoE3		14.96 ± 13.04	
apoE4		12.86 ± 9.09	
Sum of apoE		42.97 ± 28.25	
apoE2-apoE4 ratio (apoE2/apoE4)		1.48 ± 1.28	
Proportion of apoE2 in sum of apoE (apoE2%)	%	35.01 ± 16.91	
Proportion of apoE3 in sum of apoE (apoE3%)	%	32.68 ± 16.91	
Proportion of apoE4 in sum of apoE (apoE4%)	%	32.40 ± 14.12	
ARG		2.81 ± 3.12	
iNOS		2.85 ± 5.23	

**Fig. 4:**
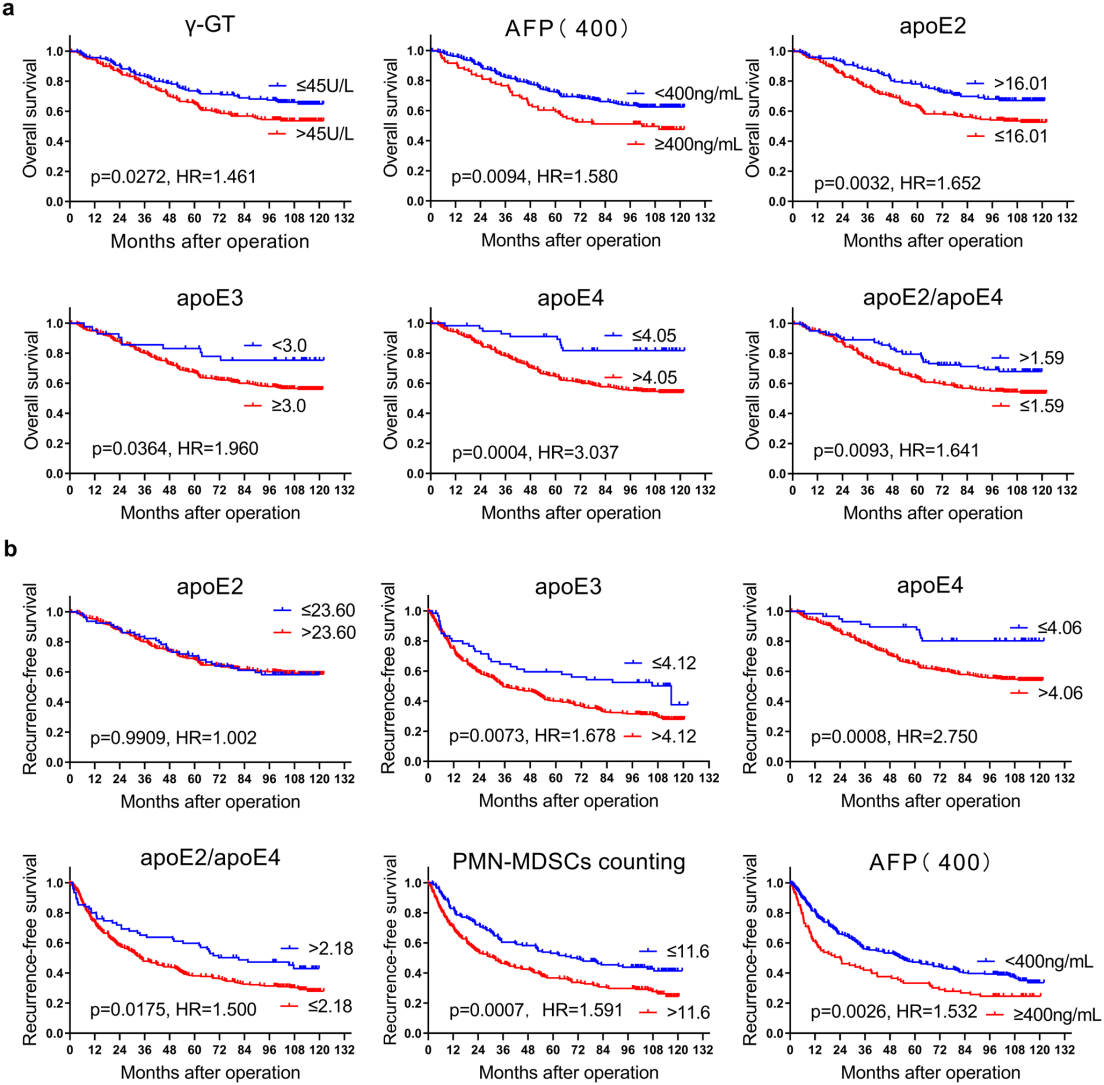
(**a**) Kaplan-Meier survival curves for OS; (**b**) Kaplan-Meier survival curves for RFS

The multivariate COX regression analysis of OS showed that, serum TB levels (HR = 3.331, 95% CI: 1.348–8.230, *p* = 0.009), γ-GT (HR = 2.916, 95% CI: 1.438–5.915, *p* = 0.003), apoE2 (HR = 6.140, 95% CI: 2.543–14.83, *p* = 0.00005), and apoE4 (HR = 7.001, 95% CI: 1.620–30.25, *p* = 0.009) in IHC staining were independent prognostic factors for OS (Table 2). The Kaplan-Meier (K-M) survival curves showed that patients with an H-score ratio of apoE2 to apoE4 higher than 1.59 had longer OS. K-M curves for main variables are presented in Fig. 4a, others are in supplementary materials (supplementary Fig. 3).

**Table 2:** COX regression analysis of the OS and RFS

Variate	Reference	OS				RFS			
		Univariate COX regression HR(95%CI)	*P* value	Multivariate COX regression HR(95%CI)	*P* value	Univariate COX regression HR(95%CI)	*P* value	Multivariate COX regression HR(95%CI)	*P* value
Age	year								
	≤ 67/>67	0.5467 (0.2874-1.040)	0.0657			0.844 (0.5572–1.278)	0.4233		
Gender	female/male	1.339 (0.7841–2.287)	0.2849			1.399 (0.9304–2.104)	0.1067		
Liver disease backgrounds									
Virus hepatitis									
HBsAg	-/+	0.9958 (0.5998–1.653)	0.9871			0.9958 (0.6746-1.470)	0.9832		
HBV DNA	50IU/mL					1.292 (0.9917–1.683)	0.0577	0.5650(0.3790–0.8430)	0.016
	-/+	1.190 (0.8503–1.666)	0.3100			0.9409 (0.5123–1.728)	0.8444		
Liver cirrhosis (pathology)	-/+	1.280 (1.066–1.536)	0.0080			1.226 (1.065–1.412)	0.0047	1.303(1.086–1.564)	0.004
G score	0/1, 2/3, 4	2.005 (0.8846–4.542)	0.0957			1.705 (0.9527-3.050)	0.0724		
S score	0/1, 2/3, 4	1.411 (0.9918–2.009)	0.0556			1.162(1.056–1.280)	0.0021		
Liver cirrhosis nodules	cm	1.721 (1.053–2.814)	0.0304			1.016 (0.6522–1.583)	0.9443		
Steatosis (pathology)	-/+	0.9219 (0.6377–1.333)	0.6653			1.023 (0.7702–1.359)	0.8745		
Tumour pathology									
Tumour counting	1/>1	1.627 (1.111–2.382)	0.0124			1.764 (1.302–2.391)	0.0002		
Sum of diameters of the tumours	cm								
	≤ 5.0cm/>5.0cm	1.619 (1.155–2.271)	0.0052			1.554 (1.191–2.028)	0.0012	1.860(1.248–2.774)	0.002
Grade of differentiation	High/Medium/Low	0.9062 (0.1262–6.509)	0.9220			1.844 (0.2578–13.19)	0.5421		
Tumour capsule	Complete/None or uncomplete	1.248 (0.8877–1.755)	0.2024			1.084 (0.8254–1.425)	0.5605		
Gross tumour emboli	-/+	0.8033 (0.2559–2.522)	0.7075			1.670 (0.8247–3.383)	0.1542		
Microscopic tumour emboli	-/+	1.289 (0.9209–1.805)	0.1389			1.264 (0.9715–1.645)	0.0811		
Laboratory examination									
Liver function test and metabolism									
Protein metabolism: Albumin	g/L								
	< 35/35–55/>55	2.069 (1.089–3.930)	0.0264			1.415 (0.7936–2.525)	0.2392		
Bilirubin metabolism test									
TB	µmol/L								
	< 3.4/3.4–20.4/>20.4	1.645 (0.9473–2.856)	0.0771	3.331(1.348–8.230)	0.009	1.291 (0.7978–2.089)	0.2983		
DBIL	µmol/L								
	≤ 6.8/>6.8	1.463 (0.9349-2.29)	0.0958			1.623 (1.135–2.319)	0.0079	1.777(1.070–2.950)	0.026
Enzymology test									
ALT	U/L								
	< 7/7–40/>40	1.01 (0.7171–1.421)	0.9564			1.110 (0.8503-1.45)	0.4418		
AST	U/L								
	< 13/13–35/>35	1.434 (1.025–2.005)	0.0352			1.538 (1.182–2.002)	0.0013	1.586(1.092–2.304)	0.016
γ-GT	U/L	1.462 (1.042–2.052)	0.0281	2.916(1.438–5.915)	0.003	1.503 (1.155–1.955)	0.0024		
Glycometabolism: blood glucose	mmol/L								
	< 3.9/3.9–5.6/>5.6	1.098 (0.7668–1.571)	0.6111			1.01 (0.7548–1.352)	0.9453		
Coagulation function									
PT	s								
	< 10.0/10.0–13.0/>13.0	0.8818 (0.5765–1.349)	0.5618			1.084 (0.7892–1.489)	0.6178		
INR		1.027 (0.4206–2.507)	0.9534			0.9670 (0.4775–1.958)	0.9257		
Lipid metabolism									
Triglyceride (TG)	mmol/L	0.7015 (0.4100–1.200)	0.1958						
	< 1.70/≥1.70					0.7507 (0.4983–1.131)	0.1703		
Cholesterol (CHO)	mmol/L								
	< 5.20/>5.20	1.037 (0.6152–1.748)	0.8912			1.279 (0.8649–1.891)	0.2177		
HDL	%								
	> 1.04/≤1.04	1.027 (0.7075–1.491)	0.8878			0.9558 (0.7110–1.285)	0.7644		
N-HDL	mg/L								
	< 4.10/≥4.10	0.9463 (0.5230–1.712)	0.8552			1.057 (0.6742–1.658)	0.8081		
LDL	mmol/L								
	< 3.4/≥3.4	1.127 (0.6230–2.040)	0.6919			1.115 (0.7041–1.767)	0.6420		
apoA I	g/L								
	< 1.0/1.0-1.9/>1.9	0.8749 (0.6051–1.265)	0.4777			0.8049 (0.6004–1.079)	0.1468		
apoB	g/L								
	< 0.75/0.75–1.50/>1.50	1.202 (0.8574–1.685)	0.2858			1.229 (0.9421–1.602)	0.1285		
apoE	mg/L								
	< 29/29–53/>53	1.195 (0.8895–1.605)	0.2370			1.035 (0.8216–1.303)	0.7723		
LPA	ng/mL								
	≤ 300/>300	0.9897 (0.6210–1.577)	0.9652			0.9494 (0.6554–1.375)	0.7835		
Liver fibrosis marker: type III procollagen	ng/mL	1.025 (0.6853–1.534)	0.9028			1.057 (0.7737–1.444)	0.7281		
	≤ 15/>15								
Tumour biomarkers									
AFP	ng/mL								
	< 20/≥20								
	< 400/≥400	1.228 (0.8521–1.769)	0.2708	1.885(0.9780–3.632)	0.058	1.275 (0.9585–1.695)	0.0951		
CA19-9	U/mL								
	< 34/≥34	1.210 (0.7440–1.968)	0.4422			0.8489 (0.5631-1.280)	0.4340		
CEA	ng/mL								
	< 5/≥5	0.7514 (0.3675–1.536)	0.4336			0.7075 (0.4115–1.217)	0.2109		
CRP	/uL								
	≤ 3.0/>3.0	0.8692 (0.5708–1.324)	0.5135			0.9663 (0.7011–1.332)	0.8340		
IHF (by H-score)									
PMN-MDSCs counting		1.360 (0.9641–1.918)	0.0798			1.551 (1.184–2.032)	0.001437	3.762(2.097–6.749)	0.00000887
PMN-MDSCs proportion	%	1.238 (0.8845–1.733)	0.2134			1.386 (1.072–1.793)	0.01272		
M-MDSCs counting		1.272 (0.9160–1.766)	0.1509			1.314 (1.014–1.703)	0.038659		
M-MDSCs proportion	%	1.343(0.9640–1.870)	0.0810			1.538 (1.179–2.008)	0.00153	2.139(1.470–3.112)	0.00007
PMN-MDSCs-M-MDSCs ratio (PMN-MDSCs/M-MDSCs)		1.290 (0.9086–1.831)	0.1545			1.397 (1.061–1.841)	0.0174	0.4540(0.2580–0.7990)	0.006
IHC (by H-score)									
apoE2		1.653 (1.161–2.353)	0.0053	6.140(2.543–14.83)	0.00005	0.7350 (0.545–0.9920)	0.0440		
apoE3		1.961 (1.031–3.729)	0.0400			1.696 (1.155–2.491)	0.0070		
apoE4		3.041 (1.599–5.784)	0.0007	7.001(1.620–30.25)	0.009	1.704 (1.161–2.502)	0.0065	1.787(1.017–3.140)	0.044
Sum of apoE		1.588 (0.9799–2.573)	0.0605			1.400 (1.071–1.830)	0.0139	1.477(1.014–2.151)	0.042
apoE2-apoE4 ratio (apoE2/apoE4)		1.642 (1.126–2.397)	0.0101			1.431 (1.027–1.994)	0.0343		
Proportion of apoE2 in sum of apoE (apoE2%)	%	1.362 (0.8487–2.184)	0.2006			1.287 (0.9950–1.666)	0.0550		
Proportion of apoE3 in sum of apoE (apoE3%)	%	1.456 (1.046–2.027)	0.0260			1.326 (1.024–1.717)	0.0325		
Proportion of apoE4 in sum of apoE (apoE4%)	%	1.523 (1.081–2.144)	0.0161			1.280 (0.9290–1.763)	0.1314		
ARG		2.312 (1.374–3.889)	0.0016			0.819 (0.6320–1.061)	0.1305		
iNOS		1.383 (0.9811–1.949)	0.0642			0.7560 (0.5810–0.9850)	0.038264		

The multivariate COX regression analysis for RFS revealed that HBV DNA (HR = 0.565, 95% CI: 0.379–0.843, *p* = 0.016), liver fibrosis (HR = 1.303, 95% CI: 1.086–1.564, *p* = 0.004), total maximum tumour diameter (> 5.3 cm, HR = 1.860, 95%CI: 1.248–2.774, *p* = 0.002), serum direct bilirubin levels (HR = 1.777, 95% CI: 1.070–2.950, *p* = 0.026), AST (HR = 1.586, 95% CI = 1.092–2.304, *p* = 0.016), apoE4 H-score in HCC tissue (> 4.06, HR = 1.787, 95% CI: 1.017–3.140, *p* = 0.044), total apoE H-score (> 53.46, HR = 1.477, 95% CI: 1.014–2.151, *p* = 0.042), PMN-MDSCs counts (> 11.6/spot, HR = 3.762, 95% CI: 2.097–6.749, *p* = 0.00000887), the proportion of M-MDSCs to total cells (< 89.32%, HR = 2.139, 95% CI: 1.470–3.112, *p* = 0.00007) and the ratio of the counts (HR = 0.454, 95% CI: 0.258–0.799, *p* = 0.006) were both independent prognostic risk factors for RFS (Table 2). Although multivariate COX regression analysis failed to obtain results with statistical significance at *p* < 0.05, trends of difference still could be seen in the survival curves for H-score of apoE3 (> 4.12), apoE2/apoE4 (≤ 2.18) and iNOS (≤ 2.07). The K-M curves of main variables are in Fig. 4b, others are in the supplementary materials (supplementary Fig. 3).

## Discussion

Recently, it was found that the relationship between lipid metabolism and immune state was capable of affecting the progress of the malignancy and response to immunotherapy in melanoma patients (Ostendorf et al. 2020). However, there is no data to indicate whether this conclusion can be applied to the liver, one of whose core functions is metabolism. In addition, liver is a tolerogenic organ, its immune status is opposite to that possessed by the skin. Phenomenologically, the vast majority of HCC occurs on the basis of liver fibrosis, which is a common pathological result of chronic liver diseases such as chronic HBV and HCV infection, metabolic dysfunction-associated steatotic liver disease (MASLD) and alcoholic liver disease (ALD), etc(Fattovich et al. 2004). Abnormal lipid metabolism is one of the shared mechanisms in the transition from many chronic liver diseases to liver fibrosis and liver cancer(Paul et al. 2022).

As one of the important molecules in the lipid metabolism pathways, apoE has been found to have key roles in various liver diseases leading to HCC. For instance, apoE is an essential composition of mature HCV particle engaging in the infection process and able to protect the virus from immune clearance(Fukuhara et al. 2014; Hueging et al. 2014; Lavie and Dubuisson 2017). In addition, low apoE expression is associated with malignant transformation of hepatocytes (Hirao-Suzuki et al. 2018), and decreased apoE levels in peripheral blood was found to be related to the metastasis of HCC (Fu et al. 2009; Yokoyama et al. 2006). These studies suggest that apoE and its associated biomolecules play multiple roles in the pathogenesis of various liver diseases and in the progression to cirrhosis and HCC in an extremely complex manner (Nascimento et al. 2020).

Liver is also well-known for its unique immune characteristics in the co-existence of clearance of pathogens and tolerance of macromolecular antigens from gut (Gao 2016; Heymann and Tacke 2016; Meijenfeldt and Jenne 2020; Stamataki and Swadling 2020). The immune suppression and tolerance make it the most easily accepted xenograft in transplantation (Inal 2014), and increases the success rate of other organs when transplanted together (Bogdanos et al. 2013). In addition, a sustained inflammatory response, including infectious inflammation and aseptic lipid inflammation leading to inflammatory-cancerous transformation is one of the most important mechanisms in the development of liver cancer (Heymann and Tacke 2016; Robinson et al. 2016; Yang et al. 2019).

Melanoma patients’ apoE subtypes are found strongly associated with disease progression and efficacy of immunotherapy, which is considered as the first-time demonstration that pre-existing genes in the genome can influence malignant progression and patients’ response to immunotherapy(Ostendorf et al. 2020). Given the metabolic function of the liver, it is a question worth exploring how apoE is involved in the immunosuppressive phenotype of HCC and affects patients’ prognosis.

To investigate the effect of apoE levels in HCC patients’ immune system, we firstly built a cohort containing 31 patients with HCC and performed flow cytometry to analyse their immune cell subsets in the peripheral blood. The results showed that the abundance of immunosuppressive cells in tumour immunity, such as CD11b^+^LOX^−^ neutrophils, CD14^+^HLA-DR^−^ M-MDSCs, CD11b^+^CD33^+^CD14^−^HLA-DR^−^ e-MDSCs and CD14^+^HLA-DR^+^ monocytes, was significantly higher in the group with low levels of apoE; also, the abundance of CD3^+^CD4^+^PD-1^+^ T lymphocytes was significantly higher in the abnormal level apoE patient groups than in the normal level apoE group, while the abundance of CD3^+^CD8^+^CTLA-4^+^ T lymphocytes was significantly higher in the low level apoE group than in the normal level apoE group. These results suggested that apoE is related to the immunosuppressive status in patients with HCC, increasing the abundance of peripheral blood MDSCs and lymphocytes expressing suppressive molecules such as PD-1 and CTLA-4. This on the one hand reflects the potential of apoE as a biomarker to assess the immune status of patients and predict their response to immunotherapies such as anti-PD-1/PD-L1 and anti-CTLA-4 antibodies, and on the other hand indicates the potential of therapeutic strategies that target apoE to regulate the immune status of patients. The application of the antibodies of apoE has been reported to enhance the effect of anti-PD-1 antibody immunotherapy in the animal models of colorectal cancer, gastric cancer and HCC(Hui et al. 2022). However, the anti-PD-1/PD-L1 or anti-CTLA-4 antibodies were not approved for HCC patients in 2012, therefore we were not able to analyse the relationship of the response to immunotherapy and apoE in HCC patients, which was a limitation of this research.

Although the results of flow cytometry preliminarily suggested the association between apoE and immunosuppressive cells in blood, it has been reported that there are more CD8^+^ T lymphocytes and CD56^+^ NK cells but fewer CD4 ^+^ T lymphocytes in liver(Norris et al. 1998; Xystrakis et al. 2015; Yuksel, Demirbas, et al., 2022), indicating the diverse phenotype profiles inside and outside livers. Therefore, to explore its application value in clinical practice, we built the second cohort to evaluate the subtypes and expression of apoE in HCC tissues. Among the results of this study, OS was better in the group with higher apoE2 expression in tumour tissue, while apoE4 was a risk factor for OS in patients with HCC. As a heterozygote can express both apoE2 and apoE4, we also analysed the impact of the intensity ratio of apoE2 to apoE4 expressed in tumour tissue on patients’ prognosis. The result that the cut-off for the value of apoE2/apoE4 was not 1, not only demonstrated the different impact of two subtypes of apoE in HCC, but also reflected the different strength of apoE2 and apoE4 on HCC patients’ prognosis. However, this finding is contrary to the findings in melanoma, where apoE4 carriers have slower tumour progression and metastasis, and a stronger response to immunotherapy(Ostendorf et al. 2020). Currently, there is still no universal, accepted systematic theory of immune escape of tumours (Bruttel and Wischhusen 2014; Hanahan 2022; Hanahan and Weinberg 2011; Villalba et al. 2013). This finding in HCC, in contrast to that in melanoma, is of great importance, the different roles of apoE2 and apoE4 in HCC and melanoma may suggest that the organ difference of tumorigenesis results in different immune mechanisms, which may partly account for the reasons why it is currently difficult to construct a widely accepted systematic theory in the immune escape of cancers. Besides, the property of apoE in regulating the inflammation could be another possible hypothesis for the contradiction(Li et al. 2015; Vitek et al. 2009). It has been reported that apoE plays a critical role in inflammatory process, in which the properties of anti-oxidation and anti-inflammation of apoE2, apoE3 and apoE4 successively decrease(Jofre-Monseny et al. 2008; Shen et al. 2023). The individuals with apoE4 usually present stronger inflammation responses than those with apoE3 (generally considered as the wild type of apoE), showing higher level of pro-inflammatory cytokines such as TNFα, IL-6 and IL-12p40(Vitek et al. 2009). And the key role that inflammation plays in the occurrence and development of HCC has been widely accepted for a long time(Galun 2016). The lower count of PMN-MDSCs and the higher proportion of M-MDSCs in the total cell count in tumour tissues showed a tendency related to longer OS. Similarly, the sum of H-scores for apoE pronounced trend in the K-M survival curves, with shorter OS in those with higher apoE expression in tumour tissue. These tendencies were consistent with past reports in the literatures, and thus correlations with statistically significant differences will be likely to be found if a larger sample size study is conducted in the future. Besides, the effects of the amount of apoE protein in tumour tissues on the OS of patients has been discussed in different cancer types before(Bancaro et al. 2023; Chen et al. 2013; Nan et al. 2021). Therefore, the result of this study suggests its potential as a possible tumour biomarker that can be used for accurate molecular diagnosis of liver cancer and prediction of patients’ OS, and may also provide a potential strategy to enhance the effect of immunotherapy for HCC. A drug design strategy called ‘small molecule structure correctors’ has been reported, through which could convert the function of apoE4 into apoE3-like. This strategy was originally developed in neurology in vitro(Chen et al. 2012), but it could be a replenishment in immunotherapy plan for HCC, after determining the genotype of apoE in patients, shedding light on personal and precise medicine for HCC. Furthermore, in the survival analysis of OS, higher γ-GT level and AFP level in peripheral blood were both significantly associated with shorter OS, consistent with the effect of both on the prognosis of patients with HCC as reported in the past(Tian et al. 2021).

In the survival analysis of RFS, higher PMN-MDSCs count and lower proportion of M-MDSCs were both significantly associated with shorter RFS. Although multivariate COX regression analysis failed to obtain results with statistical significance, differences could be seen in the survival curves for H-score of apoE3, H-score of apo4, H-score ratio of apoE2 and apoE4, total apoE H-score and H-score of iNOS in tumour tissues, suggesting that these indicators were associated with recurrence of HCC and also consistent with the trend of OS. Conversely, the H-scores of apoE2 and ARG showed no significant effect on RFS in this study. Among other clinical tests and pathological variables in this study, RFS was significantly shorter in those with a greater total maximum tumour diameter and those with moderate and severe cirrhosis compared to those without cirrhosis, while RFS in patients with mild cirrhosis was close to that of those without cirrhosis. Among the variables related to lipid metabolism, it is particularly noteworthy that RFS was significantly shorter in patients with higher N-HDL level. N-HDL comprises the sum of lipoproteins other than HDL, and was introduced to the academic community as an indicator of cardiovascular disease at first, but has recently gained attention for its value and clinical significance in oncology(Guaita-Esteruelas et al. 2017; Kong et al. 2022). The effect of elevated N-HDL on patients’ OS reflects the complexity of lipid metabolism in HCC, and the mechanisms through which different types of lipoproteins are involved in the development of HCC, the body’s anti-tumour immune response and the immune escape process of HCC are particularly worthy of further investigation.

## Conclusion

The lower level of apoE in peripheral blood was associated with the immunosuppressive status in HCC patients. The high expression of apoE2 was a protective factor for patients’ OS, while patients with high expression of apoE4 subtype had shorter OS; the density of infiltrated PMN-MDSCs in tumour tissues was negatively correlated with patients’ OS and RFS, suggesting that it was related to patients’ tumour recurrence and poor prognosis.

In addition, the striking finding of this study is that the prognostic impact of different apoE subtypes on HCC is opposite to that in melanoma, emphasizing the variability and complexity in the mechanisms of tumour immune escape among different cancer species and organ backgrounds.

## Electronic supplementary material

Below is the link to the electronic supplementary material.

Supplementary Material 1

## Data Availability

All data that support the findings of this study are available from the corresponding authors upon reasonable request.

## Abbreviations

HCC: Hepatocellular carcinoma
apoE: apolipoprotein E
MDSC: Myeloid-derived suppressor cell
PMN-MDSCs: Polymorphonuclear granulocyte-like MDSCs
M-MDSCs: Monocyte-like MDSCs
ARG: Arginase
iNOS: Inducible nitric oxide synthase
OS: Overall survival
RFS: Recurrence-free survival
PLC: Primary liver cancer
HBV: Hepatitis B virus
HCV: Hepatitis C virus
CT: Computed tomography
MRI: Magnetic resonance imaging
FFPE: Formalin-fixed paraffin-embedded
IHC: Immunohistochemistry
IHF: Immunofluorescence
HR: Hazard ratio
CI: Confidence interval
TB: Total bilirubin
D-BIL: Direct bilirubin
ALT: Alanine transaminase
AST: Aspartate transaminase
PT: Prothrombin time
INR: International normalized ratio
TG: Triglyceride
CHO: Cholesterol
HDL: High density lipoprotein
LDL: Low density lipoprotein
LPA: Lipoprotein A
AFP: Alpha-fetoprotein
CEA: Carcinoembryonic antigen
CRP: C reactive protein
γ-GT: Gamma-glutamyl transpeptidase
