# Supplementary material for: Effects of the subtypes of apolipoprotein E on immune inhibition and prognosis in patients with Hepatocellular Carcinoma
Source: J Cancer Res Clin Oncol. 2024 Jul 8;150(7):341. doi: 10.1007/s00432-024-05856-6 (PMC11230970; doi:10.1007/s00432-024-05856-6)
Supplement: Supplementary file 1 — Supplementary Material 1 [file 432_2024_5856_MOESM1_ESM.docx]

Supplementary Figures


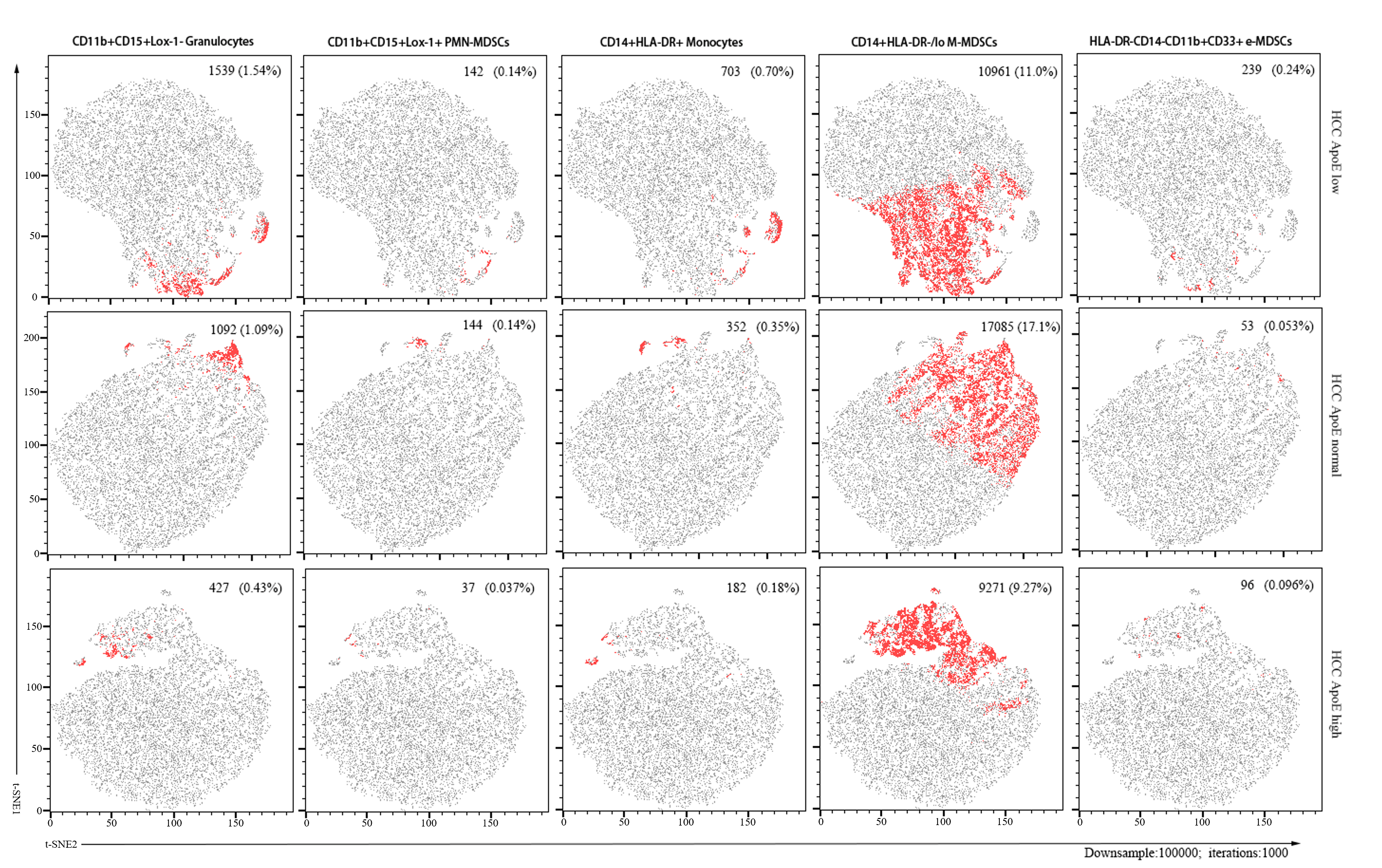


Supplementary figure 1. The abundance of multiple types of immune suppressor cells in HCC categorized into the subgroups of high, medium and low levels of apoE in peripheral blood.


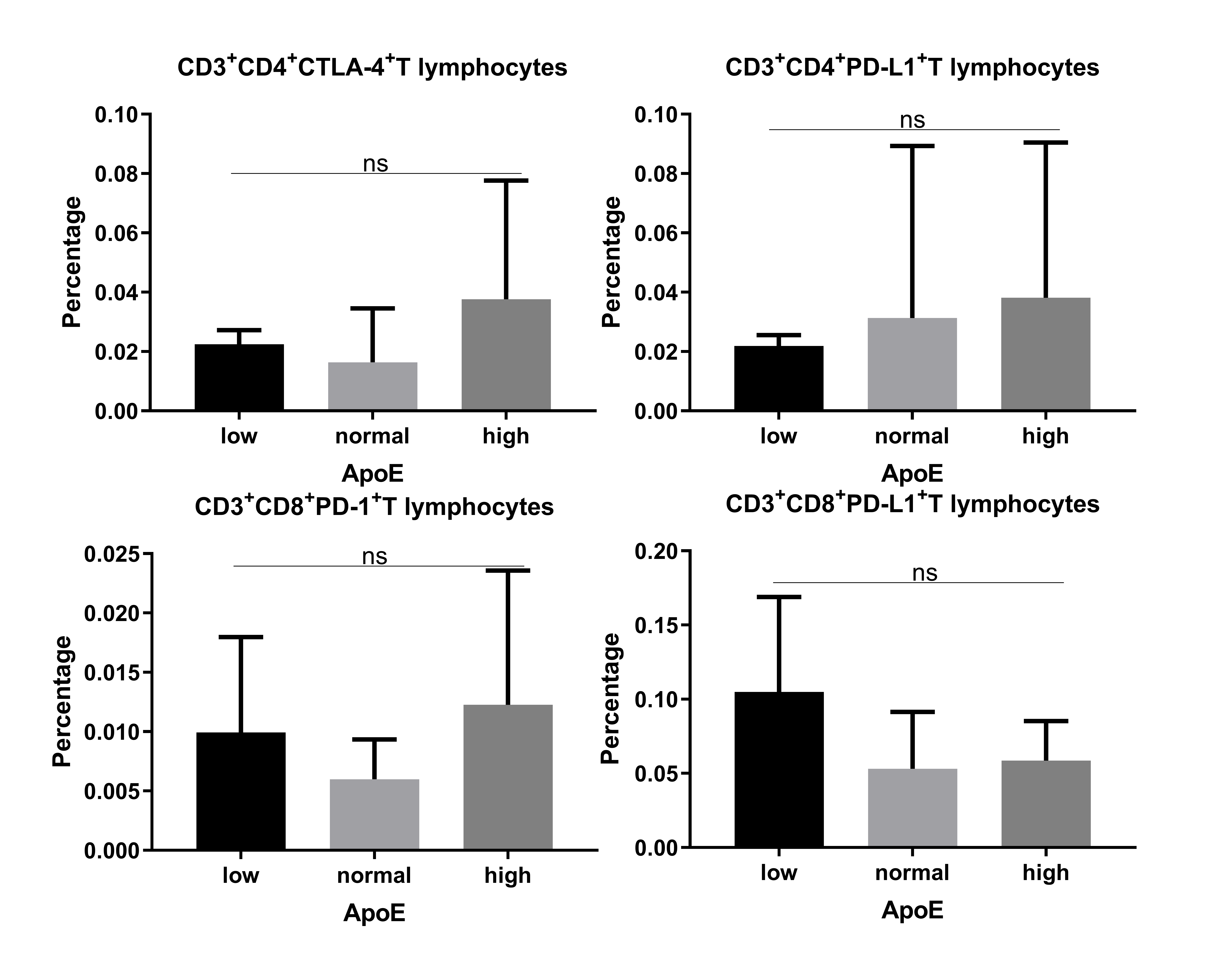


Supplementary figure 2. The abundance of T lymphocytes subgroups with expressions of immune suppressor biomarkers in HCC patients with low, normal or high levels of apoE.


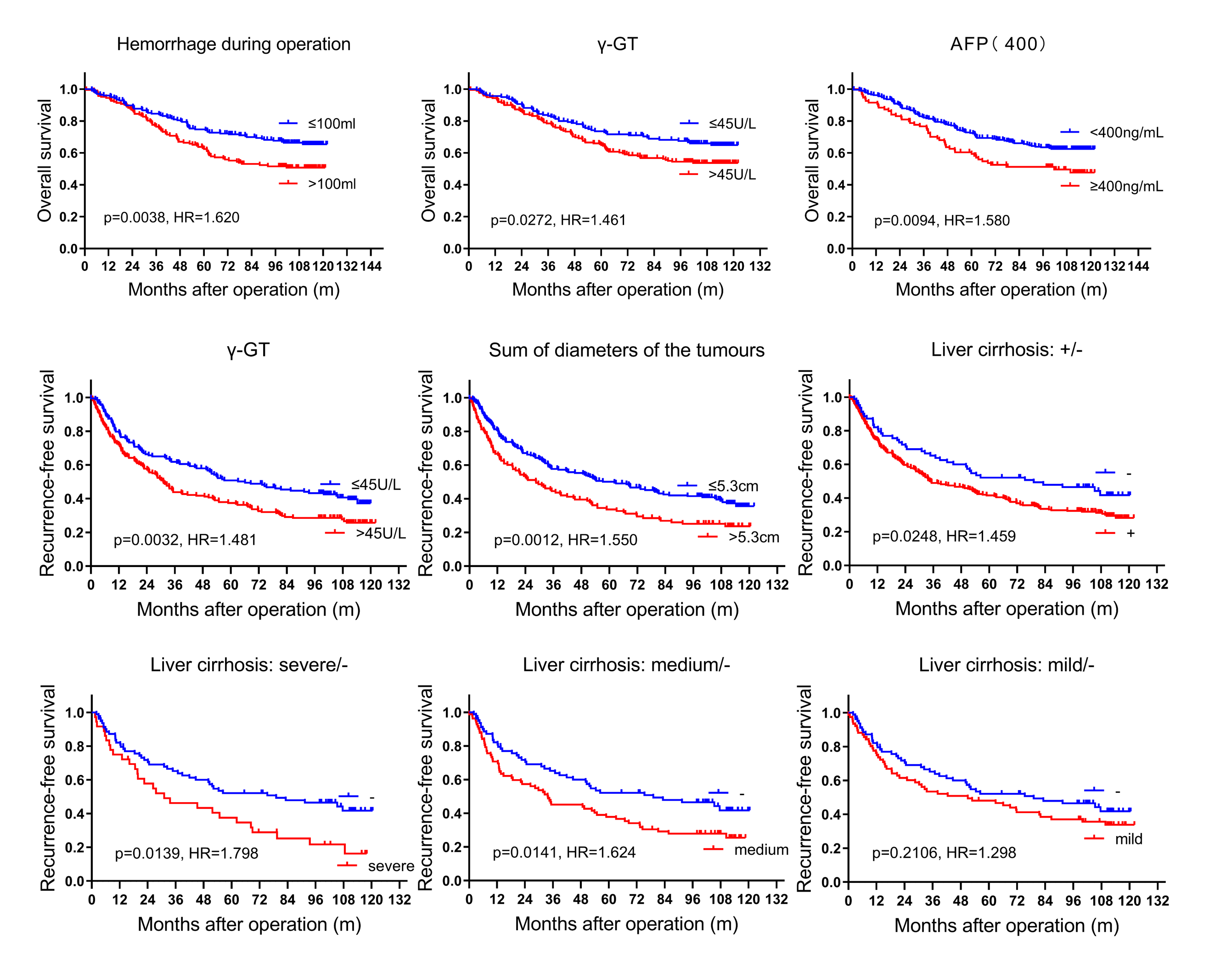


Supplementary figure 3. OS and RFS K-M survival curves.
